# Dichlorido{1-[*N*-(5-chloro-2-oxidophen­yl)carboximido­yl]naphthalen-2-olato-κ^3^ *O*,*N*,*O*′}(methanol-κ*O*)tin(IV)

**DOI:** 10.1107/S160053681200390X

**Published:** 2012-02-04

**Authors:** Gholam Hossein Shahverdizadeh, Seik Weng Ng, Edward R. T. Tiekink, Babak Mirtamizdoust

**Affiliations:** a Department of Chemistry, Faculty of Science, Tabriz Branch, Islamic Azad University, PO Box 1655, Tabriz, Iran; b Department of Chemistry, University of Malaya, 50603 Kuala Lumpur, Malaysia; c Chemistry Department, Faculty of Science, King Abdulaziz University, PO Box 80203 Jeddah, Saudi Arabia; d Department of Inorganic Chemistry, Faculty of Chemistry, University of Tabriz, PO Box 5166616471, Tabriz, Iran

## Abstract

In the title complex, [Sn(C_17_H_10_ClNO_2_)Cl_2_(CH_3_OH)], the Sn^IV^ atom features a distorted octa­hedral geometry defined by the *O*,*N*,*O*′-donors of the dianion, two Cl atoms and the methanol O atom. The six-membered chelate ring has a half-chair conformation with the Sn atom lying 0.449 (4) Å out of the plane defined by the remaining atoms (r.m.s. deviation = 0.0238 Å). Supra­molecular helical chains along [100], mediated by O—H⋯O hydrogen bonds, feature in the crystal packing. Chains are linked by C—H⋯O, C—H⋯Cl and π–π [centroid–centroid distance = 3.598 (2) Å] inter­actions.

## Related literature

For background to related Sn(IV) Schiff base compounds and a closely related structure, see: Pettinari *et al.* (2001 ▶). For specialized crystallization techniques, see: Harrowfield *et al.* (1996 ▶).
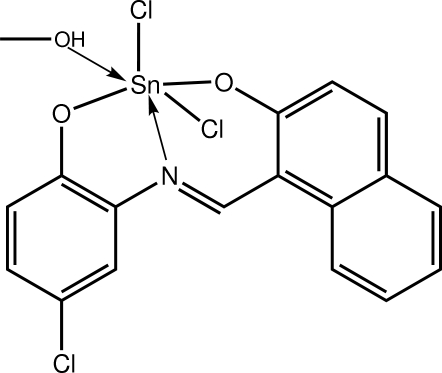


## Experimental

### 

#### Crystal data

[Sn(C_17_H_10_ClNO_2_)Cl_2_(CH_4_O)]
*M* *_r_* = 517.34
Orthorhombic, 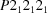

*a* = 9.9767 (3) Å
*b* = 11.1639 (3) Å
*c* = 16.2755 (5) Å
*V* = 1812.75 (9) Å^3^
*Z* = 4
Mo *K*α radiation
μ = 1.87 mm^−1^
*T* = 100 K
0.25 × 0.20 × 0.15 mm

#### Data collection

Agilent SuperNova Dual diffractometer with an Atlas detector
Absorption correction: multi-scan (*CrysAlis PRO*; Agilent, 2010 ▶) *T*_min_ = 0.819, *T*_max_ = 1.000
6571 measured reflections
4139 independent reflections
3913 reflections with *I* > 2σ(*I*)
*R*_int_ = 0.024

#### Refinement

*R*[*F* ^2^ > 2σ(*F* ^2^)] = 0.030
*wR*(*F* ^2^) = 0.059
*S* = 1.01
4139 reflections
239 parameters
1 restraint
H atoms treated by a mixture of independent and constrained refinement
Δρ_max_ = 0.54 e Å^−3^
Δρ_min_ = −0.73 e Å^−3^
Absolute structure: Flack (1983 ▶), 1765 Friedel pairs
Flack parameter: −0.036 (19)

### 

Data collection: *CrysAlis PRO* (Agilent, 2010 ▶); cell refinement: *CrysAlis PRO*; data reduction: *CrysAlis PRO*; program(s) used to solve structure: *SHELXS97* (Sheldrick, 2008 ▶); program(s) used to refine structure: *SHELXL97* (Sheldrick, 2008 ▶); molecular graphics: *ORTEP-3* (Farrugia, 1997 ▶) and *DIAMOND* (Brandenburg, 2006 ▶); software used to prepare material for publication: *publCIF* (Westrip, 2010 ▶).

## Supplementary Material

Crystal structure: contains datablock(s) global, I. DOI: 10.1107/S160053681200390X/hg5171sup1.cif

Structure factors: contains datablock(s) I. DOI: 10.1107/S160053681200390X/hg5171Isup2.hkl

Additional supplementary materials:  crystallographic information; 3D view; checkCIF report

## Figures and Tables

**Table 1:** Selected bond lengths (Å)

Sn—Cl1	2.3398 (10)
Sn—Cl2	2.3807 (9)
Sn—O1	2.050 (2)
Sn—O2	2.010 (3)
Sn—O3	2.174 (3)
Sn—N1	2.144 (3)

**Table 2:** Hydrogen-bond geometry (Å, °)

*D*—H⋯*A*	*D*—H	H⋯*A*	*D*⋯*A*	*D*—H⋯*A*
O3—H3O⋯O1^i^	0.84 (1)	1.80 (1)	2.633 (4)	174 (4)
C2—H2⋯O2^ii^	0.95	2.50	3.363 (4)	151
C16—H16⋯Cl3^iii^	0.95	2.74	3.542 (4)	143
C18—H18*A*⋯Cl2^i^	0.98	2.77	3.707 (4)	161

Symmetry codes: (i) 
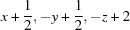
; (ii) 
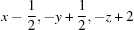
; (iii) 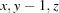.

**Fig. 1.**
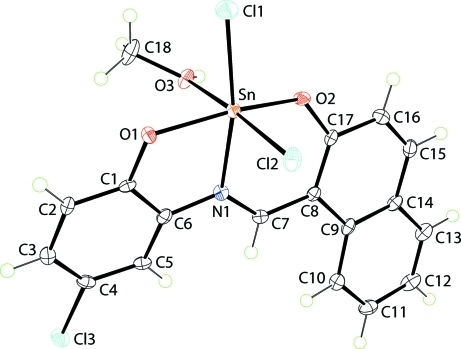
The molecular structure of (I) showing the atom-labelling scheme and displacement ellipsoids at the 70% probability level.

**Fig. 2.**
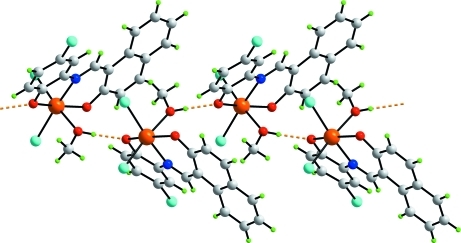
View of the supramolecular chain along [100] in (I) mediated by O—H···O hydrogen bonding shown as orange dashed lines.

**Fig. 3.**
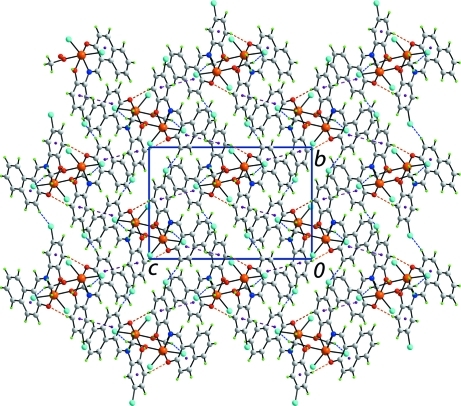
A view in projection down the a axis of the unit-cell contents of (I). The C—H···O, C—H···Cl and π–π interactions are shown as orange, blue and purple dashed lines.

## supplementary crystallographic information

### Comment

Original interest in tin(IV) compounds with Schiff bases ligands based on the 2-{[(2-hydroxyphenyl)imino]methyl}phenol parent compound stemmed from possible applications in medicinal chemistry (Pettinari *et al.*, 2001). This motivated the synthesis and characterization of the title compound, (I).

In (I), Fig. 1, the Sn^IV^ atom is coordinated by the tridentate, dinegative Schiff base, two Cl atoms and the O atom of a methanol molecule to define a distorted octahedral geometry within a Cl_2_NO_3_ donor set, Table 1. The five-membered chelate ring is approximately planar with a r.m.s. deviation of 0.058 Å. By contrast, the six-membered chelate ring has a half-chair conformation as the Sn atom lies 0.449 (4) Å out of the plane defined by the five remaining atoms (r.m.s. deviation = 0.024 Å). The Sn—Cl2 bond length is significantly longer than that of Sn—Cl1, a difference which is correlated with the Cl2 atom being *trans* to the methanol-*O* atom.

The most significant feature of the crystal packing is the formation of helical supramolecular chains along [100] mediated by O—H···O hydrogen bonding, Fig. 2 and Table 2. Chains are consolidated in the crystal packing by C—H···O and C—H···Cl interactions. Further stability is provided by π–π contacts [ring centroid(C1–C6)···ring centroid(C8,C9,C14–C17)^i^ = 3.598 (2) Å, angle = 10.47 (17)° for *i*: 1 - *x*, 1/2 + *y*, 3/2 - *z*).

### Experimental

A solution of 2-amino-4-chlorophenol (10 mmol) in EtOH (30 ml) was added drop-wise to the solution of 2-hydroxy-1-naphthaldehyde (10 mmol) in EtOH (20 ml). The mixture was refluxed for 5 h. The yellow precipitate was removed by filtration and recrystallized from MeOH solution. The ligand (0.5 mmol) was placed in one arm of a branched tube (Harrowfield *et al.*, 1996) and tin(IV) chloride (0.5 mmol) in the other. Methanol was then added to fill both arms, the tube sealed and the ligand-containing arm immersed in a bath at 333 K, while the other was left at ambient temperature. After three weeks crystals deposited in the arm held at ambient temperature. They were filtered off, washed with acetone and ether, and air-dried. Yield: 68%. *M*.pt.: 571 K (dec.).

### Refinement

Carbon-bound H-atoms were placed in calculated positions [C—H = 0.95 to 0.98 Å and with *U*_iso_(H) = 1.2 to 1.5*U*_eq_(C)] and were included in the refinement in the riding model approximation. The hydroxy H-atom was located in a difference Fourier map and was refined with a distance restraint of O–H = 0.84±0.01 Å; *U*_iso_ was refined. The (0 1 1) reflection was omitted from the final refinement owing to poor agreement.

### Crystal data

**Table:**

[Sn(C_17_H_10_ClNO_2_)Cl_2_(CH_4_O)]	*F*(000) = 1016
*M**_r_* = 517.34	*D*_x_ = 1.896 Mg m^−^^3^
Orthorhombic, *P*2_1_2_1_2_1_	Mo *K*α radiation, λ = 0.71073 Å
Hall symbol: P 2ac 2ab	Cell parameters from 4166 reflections
*a* = 9.9767 (3) Å	θ = 2.2–27.5°
*b* = 11.1639 (3) Å	µ = 1.87 mm^−^^1^
*c* = 16.2755 (5) Å	*T* = 100 K
*V* = 1812.75 (9) Å^3^	Prism, brown
*Z* = 4	0.25 × 0.20 × 0.15 mm

### Data collection

**Table:**

Agilent SuperNova Dual diffractometer with an Atlas detector	4139 independent reflections
Radiation source: SuperNova (Mo) X-ray Source	3913 reflections with *I* > 2σ(*I*)
Mirror monochromator	*R*_int_ = 0.024
Detector resolution: 10.4041 pixels mm^-1^	θ_max_ = 27.6°, θ_min_ = 2.4°
ω scan	*h* = −12→7
Absorption correction: multi-scan (*CrysAlis PRO*; Agilent, 2010)	*k* = −10→14
*T*_min_ = 0.819, *T*_max_ = 1.000	*l* = −21→20
6571 measured reflections	

### Refinement

**Table:**

Refinement on *F*^2^	Secondary atom site location: difference Fourier map
Least-squares matrix: full	Hydrogen site location: inferred from neighbouring sites
*R*[*F*^2^ > 2σ(*F*^2^)] = 0.030	H atoms treated by a mixture of independent and constrained refinement
*wR*(*F*^2^) = 0.059	*w* = 1/[σ^2^(*F*_o_^2^) + (0.0231*P*)^2^] where *P* = (*F*_o_^2^ + 2*F*_c_^2^)/3
*S* = 1.01	(Δ/σ)_max_ = 0.002
4139 reflections	Δρ_max_ = 0.54 e Å^−^^3^
239 parameters	Δρ_min_ = −0.73 e Å^−^^3^
1 restraint	Absolute structure: Flack (1983), 1765 Friedel pairs
Primary atom site location: structure-invariant direct methods	Flack parameter: −0.036 (19)

### Special details

**Table:**

Geometry. All s.u.'s (except the s.u. in the dihedral angle between two l.s. planes) are estimated using the full covariance matrix. The cell s.u.'s are taken into account individually in the estimation of s.u.'s in distances, angles and torsion angles; correlations between s.u.'s in cell parameters are only used when they are defined by crystal symmetry. An approximate (isotropic) treatment of cell s.u.'s is used for estimating s.u.'s involving l.s. planes.
Refinement. Refinement of *F*^2^ against ALL reflections. The weighted *R*-factor *wR* and goodness of fit *S* are based on *F*^2^, conventional *R*-factors *R* are based on *F*, with *F* set to zero for negative *F*^2^. The threshold expression of *F*^2^ > 2σ(*F*^2^) is used only for calculating *R*-factors(gt) *etc*. and is not relevant to the choice of reflections for refinement. *R*-factors based on *F*^2^ are statistically about twice as large as those based on *F*, and *R*- factors based on ALL data will be even larger.

### Fractional atomic coordinates and isotropic or equivalent isotropic displacement parameters (Å^2^)

**Table:**

	*x*	*y*	*z*	*U*_iso_*/*U*_eq_	
Sn	0.39500 (3)	0.17557 (2)	0.915201 (14)	0.01147 (6)	
Cl1	0.27998 (11)	0.03339 (8)	0.99384 (6)	0.0222 (2)	
Cl2	0.27645 (10)	0.15018 (8)	0.78945 (5)	0.0197 (2)	
Cl3	0.50466 (11)	0.79039 (7)	0.89690 (6)	0.0188 (2)	
O1	0.2770 (2)	0.3133 (2)	0.95636 (14)	0.0121 (5)	
O2	0.5532 (3)	0.0747 (2)	0.88264 (15)	0.0146 (6)	
O3	0.5167 (3)	0.2113 (2)	1.02329 (15)	0.0149 (6)	
H3O	0.5989 (13)	0.198 (3)	1.029 (2)	0.021 (11)*	
N1	0.5035 (3)	0.3262 (3)	0.86761 (16)	0.0119 (6)	
C1	0.3288 (4)	0.4240 (3)	0.9413 (2)	0.0113 (8)	
C2	0.2641 (4)	0.5253 (3)	0.9715 (2)	0.0112 (7)	
H2	0.1836	0.5175	1.0023	0.013*	
C3	0.3185 (4)	0.6384 (3)	0.9563 (2)	0.0137 (8)	
H3	0.2741	0.7083	0.9756	0.016*	
C4	0.4377 (4)	0.6482 (3)	0.9128 (2)	0.0136 (7)	
C5	0.5029 (4)	0.5491 (3)	0.8821 (2)	0.0124 (8)	
H5	0.5840	0.5580	0.8521	0.015*	
C6	0.4490 (4)	0.4361 (3)	0.8953 (2)	0.0123 (8)	
C7	0.6067 (4)	0.3186 (3)	0.81737 (18)	0.0125 (7)	
H7	0.6401	0.3918	0.7958	0.015*	
C8	0.6736 (4)	0.2118 (3)	0.7921 (2)	0.0110 (7)	
C9	0.7848 (4)	0.2219 (3)	0.7340 (2)	0.0133 (8)	
C10	0.8181 (4)	0.3302 (4)	0.6923 (2)	0.0144 (7)	
H10	0.7644	0.3995	0.7006	0.017*	
C11	0.9261 (4)	0.3361 (3)	0.6407 (2)	0.0189 (8)	
H11	0.9456	0.4093	0.6135	0.023*	
C12	1.0089 (4)	0.2361 (3)	0.6272 (2)	0.0196 (9)	
H12	1.0856	0.2423	0.5929	0.023*	
C13	0.9775 (4)	0.1303 (3)	0.6639 (2)	0.0166 (8)	
H13	1.0317	0.0619	0.6538	0.020*	
C14	0.8661 (4)	0.1203 (3)	0.7168 (2)	0.0138 (8)	
C15	0.8323 (4)	0.0086 (3)	0.7535 (2)	0.0172 (8)	
H15	0.8852	−0.0599	0.7413	0.021*	
C16	0.7278 (4)	−0.0026 (3)	0.8048 (2)	0.0175 (9)	
H16	0.7070	−0.0790	0.8271	0.021*	
C17	0.6474 (4)	0.0978 (3)	0.8264 (2)	0.0115 (8)	
C18	0.4697 (4)	0.2485 (4)	1.1025 (2)	0.0243 (10)	
H18A	0.5462	0.2583	1.1397	0.036*	
H18B	0.4087	0.1877	1.1247	0.036*	
H18C	0.4220	0.3249	1.0974	0.036*	

### Atomic displacement parameters (Å^2^)

**Table:**

	*U*^11^	*U*^22^	*U*^33^	*U*^12^	*U*^13^	*U*^23^
Sn	0.00915 (11)	0.01150 (10)	0.01375 (11)	−0.00087 (11)	0.00154 (11)	−0.00057 (11)
Cl1	0.0196 (5)	0.0204 (4)	0.0265 (5)	−0.0049 (4)	0.0050 (5)	0.0019 (4)
Cl2	0.0179 (5)	0.0225 (5)	0.0187 (4)	−0.0008 (4)	−0.0027 (4)	−0.0060 (4)
Cl3	0.0204 (5)	0.0125 (4)	0.0237 (5)	−0.0033 (4)	0.0017 (4)	−0.0002 (3)
O1	0.0082 (13)	0.0103 (11)	0.0176 (12)	−0.0012 (11)	0.0042 (10)	−0.0011 (11)
O2	0.0136 (15)	0.0138 (12)	0.0166 (12)	0.0019 (10)	0.0064 (11)	0.0044 (11)
O3	0.0062 (14)	0.0246 (14)	0.0138 (12)	0.0026 (12)	0.0009 (12)	−0.0019 (11)
N1	0.0089 (15)	0.0126 (13)	0.0141 (13)	−0.0011 (15)	−0.0002 (12)	−0.0006 (14)
C1	0.012 (2)	0.0145 (16)	0.0078 (15)	−0.0021 (15)	−0.0013 (15)	0.0028 (14)
C2	0.0077 (19)	0.0168 (16)	0.0091 (16)	0.0018 (15)	−0.0013 (15)	−0.0028 (15)
C3	0.012 (2)	0.0154 (17)	0.0135 (17)	0.0014 (15)	−0.0021 (16)	−0.0020 (15)
C4	0.0153 (19)	0.0120 (16)	0.0137 (15)	−0.0025 (13)	−0.0055 (16)	0.0029 (16)
C5	0.010 (2)	0.0152 (17)	0.0124 (16)	−0.0014 (15)	−0.0013 (16)	0.0041 (15)
C6	0.0124 (19)	0.0162 (16)	0.0082 (17)	0.0005 (14)	−0.0024 (14)	−0.0002 (14)
C7	0.0123 (17)	0.0130 (14)	0.0122 (15)	−0.0007 (19)	−0.0006 (15)	0.0019 (15)
C8	0.0089 (19)	0.0152 (17)	0.0088 (16)	0.0019 (14)	−0.0014 (14)	0.0000 (14)
C9	0.011 (2)	0.0194 (17)	0.0096 (16)	−0.0009 (16)	−0.0011 (16)	−0.0028 (15)
C10	0.0099 (18)	0.0185 (17)	0.0148 (16)	0.0017 (17)	−0.0004 (14)	−0.0010 (17)
C11	0.019 (2)	0.0210 (18)	0.0165 (16)	−0.0055 (18)	0.0011 (15)	0.0042 (17)
C12	0.009 (2)	0.033 (2)	0.0164 (19)	−0.0003 (18)	0.0017 (17)	0.0039 (18)
C13	0.009 (2)	0.0265 (19)	0.0142 (17)	0.0038 (16)	0.0000 (16)	0.0002 (17)
C14	0.009 (2)	0.0210 (18)	0.0110 (16)	−0.0010 (15)	0.0012 (15)	−0.0001 (15)
C15	0.020 (2)	0.0184 (19)	0.0131 (18)	0.0062 (16)	−0.0024 (17)	−0.0034 (16)
C16	0.021 (2)	0.0137 (17)	0.0176 (18)	0.0028 (17)	0.0003 (17)	0.0024 (16)
C17	0.0099 (19)	0.0155 (16)	0.0089 (16)	0.0004 (15)	−0.0001 (14)	−0.0021 (15)
C18	0.016 (2)	0.043 (2)	0.0141 (19)	0.0063 (19)	0.0019 (17)	−0.0063 (18)

### Geometric parameters (Å, º)

**Table:**

Sn—Cl1	2.3398 (10)	C7—C8	1.427 (5)
Sn—Cl2	2.3807 (9)	C7—H7	0.9500
Sn—O1	2.050 (2)	C8—C17	1.414 (5)
Sn—O2	2.010 (3)	C8—C9	1.462 (5)
Sn—O3	2.174 (3)	C9—C14	1.422 (5)
Sn—N1	2.144 (3)	C9—C10	1.426 (5)
Cl3—C4	1.742 (3)	C10—C11	1.368 (5)
O1—C1	1.361 (4)	C10—H10	0.9500
O2—C17	1.337 (4)	C11—C12	1.406 (5)
O3—C18	1.434 (4)	C11—H11	0.9500
O3—H3O	0.840 (10)	C12—C13	1.360 (5)
N1—C7	1.318 (4)	C12—H12	0.9500
N1—C6	1.416 (5)	C13—C14	1.410 (5)
C1—C2	1.392 (5)	C13—H13	0.9500
C1—C6	1.420 (5)	C14—C15	1.423 (5)
C2—C3	1.397 (5)	C15—C16	1.342 (5)
C2—H2	0.9500	C15—H15	0.9500
C3—C4	1.388 (5)	C16—C17	1.422 (5)
C3—H3	0.9500	C16—H16	0.9500
C4—C5	1.377 (5)	C18—H18A	0.9800
C5—C6	1.388 (5)	C18—H18B	0.9800
C5—H5	0.9500	C18—H18C	0.9800
			
O2—Sn—O1	163.28 (10)	N1—C6—C1	114.2 (3)
O2—Sn—N1	87.01 (11)	N1—C7—C8	126.7 (3)
O1—Sn—N1	79.61 (11)	N1—C7—H7	116.6
O2—Sn—O3	82.99 (10)	C8—C7—H7	116.6
O1—Sn—O3	85.31 (10)	C17—C8—C7	123.5 (3)
N1—Sn—O3	82.33 (10)	C17—C8—C9	117.7 (3)
O2—Sn—Cl1	98.58 (7)	C7—C8—C9	118.5 (3)
O1—Sn—Cl1	92.78 (7)	C14—C9—C10	116.7 (3)
N1—Sn—Cl1	167.70 (8)	C14—C9—C8	119.9 (3)
O3—Sn—Cl1	87.47 (7)	C10—C9—C8	123.3 (3)
O2—Sn—Cl2	95.55 (8)	C11—C10—C9	121.0 (4)
O1—Sn—Cl2	94.87 (7)	C11—C10—H10	119.5
N1—Sn—Cl2	91.92 (8)	C9—C10—H10	119.5
O3—Sn—Cl2	174.12 (7)	C10—C11—C12	121.4 (4)
Cl1—Sn—Cl2	98.39 (4)	C10—C11—H11	119.3
C1—O1—Sn	113.8 (2)	C12—C11—H11	119.3
C17—O2—Sn	128.6 (2)	C13—C12—C11	119.0 (4)
C18—O3—Sn	126.8 (2)	C13—C12—H12	120.5
C18—O3—H3O	106 (3)	C11—C12—H12	120.5
Sn—O3—H3O	127 (3)	C12—C13—C14	121.2 (4)
C7—N1—C6	123.6 (3)	C12—C13—H13	119.4
C7—N1—Sn	124.6 (3)	C14—C13—H13	119.4
C6—N1—Sn	111.8 (2)	C13—C14—C9	120.4 (3)
O1—C1—C2	119.8 (3)	C13—C14—C15	120.8 (3)
O1—C1—C6	120.1 (3)	C9—C14—C15	118.7 (3)
C2—C1—C6	120.1 (3)	C16—C15—C14	121.8 (4)
C1—C2—C3	119.4 (3)	C16—C15—H15	119.1
C1—C2—H2	120.3	C14—C15—H15	119.1
C3—C2—H2	120.3	C15—C16—C17	121.2 (3)
C4—C3—C2	119.6 (3)	C15—C16—H16	119.4
C4—C3—H3	120.2	C17—C16—H16	119.4
C2—C3—H3	120.2	O2—C17—C8	125.0 (3)
C5—C4—C3	121.8 (3)	O2—C17—C16	114.4 (3)
C5—C4—Cl3	119.8 (3)	C8—C17—C16	120.5 (3)
C3—C4—Cl3	118.4 (3)	O3—C18—H18A	109.5
C4—C5—C6	119.4 (4)	O3—C18—H18B	109.5
C4—C5—H5	120.3	H18A—C18—H18B	109.5
C6—C5—H5	120.3	O3—C18—H18C	109.5
C5—C6—N1	126.2 (3)	H18A—C18—H18C	109.5
C5—C6—C1	119.7 (3)	H18B—C18—H18C	109.5
			
O2—Sn—O1—C1	31.6 (5)	Sn—N1—C6—C5	173.6 (3)
N1—Sn—O1—C1	−5.7 (2)	C7—N1—C6—C1	173.1 (3)
O3—Sn—O1—C1	77.3 (2)	Sn—N1—C6—C1	−5.7 (4)
Cl1—Sn—O1—C1	164.5 (2)	O1—C1—C6—C5	−178.4 (3)
Cl2—Sn—O1—C1	−96.8 (2)	C2—C1—C6—C5	1.3 (5)
O1—Sn—O2—C17	−59.1 (5)	O1—C1—C6—N1	1.0 (5)
N1—Sn—O2—C17	−22.5 (3)	C2—C1—C6—N1	−179.4 (3)
O3—Sn—O2—C17	−105.1 (3)	C6—N1—C7—C8	174.6 (3)
Cl1—Sn—O2—C17	168.6 (3)	Sn—N1—C7—C8	−6.8 (5)
Cl2—Sn—O2—C17	69.2 (3)	N1—C7—C8—C17	−8.3 (6)
O2—Sn—O3—C18	−156.5 (3)	N1—C7—C8—C9	178.5 (3)
O1—Sn—O3—C18	35.5 (3)	C17—C8—C9—C14	−2.9 (5)
N1—Sn—O3—C18	115.6 (3)	C7—C8—C9—C14	170.7 (3)
Cl1—Sn—O3—C18	−57.5 (3)	C17—C8—C9—C10	177.2 (3)
Cl2—Sn—O3—C18	127.5 (7)	C7—C8—C9—C10	−9.1 (5)
O2—Sn—N1—C7	17.5 (3)	C14—C9—C10—C11	−2.2 (5)
O1—Sn—N1—C7	−172.6 (3)	C8—C9—C10—C11	177.7 (3)
O3—Sn—N1—C7	100.8 (3)	C9—C10—C11—C12	−0.5 (5)
Cl1—Sn—N1—C7	135.0 (3)	C10—C11—C12—C13	2.5 (6)
Cl2—Sn—N1—C7	−78.0 (3)	C11—C12—C13—C14	−1.7 (6)
O2—Sn—N1—C6	−163.7 (2)	C12—C13—C14—C9	−1.0 (6)
O1—Sn—N1—C6	6.2 (2)	C12—C13—C14—C15	178.5 (4)
O3—Sn—N1—C6	−80.4 (2)	C10—C9—C14—C13	2.9 (5)
Cl1—Sn—N1—C6	−46.2 (5)	C8—C9—C14—C13	−176.9 (3)
Cl2—Sn—N1—C6	100.8 (2)	C10—C9—C14—C15	−176.7 (3)
Sn—O1—C1—C2	−175.1 (3)	C8—C9—C14—C15	3.5 (5)
Sn—O1—C1—C6	4.6 (4)	C13—C14—C15—C16	179.1 (4)
O1—C1—C2—C3	179.6 (3)	C9—C14—C15—C16	−1.3 (6)
C6—C1—C2—C3	−0.1 (5)	C14—C15—C16—C17	−1.5 (6)
C1—C2—C3—C4	−1.4 (5)	Sn—O2—C17—C8	16.4 (5)
C2—C3—C4—C5	1.7 (5)	Sn—O2—C17—C16	−166.4 (2)
C2—C3—C4—Cl3	−178.6 (3)	C7—C8—C17—O2	3.9 (6)
C3—C4—C5—C6	−0.5 (5)	C9—C8—C17—O2	177.3 (3)
Cl3—C4—C5—C6	179.8 (3)	C7—C8—C17—C16	−173.1 (3)
C4—C5—C6—N1	179.8 (3)	C9—C8—C17—C16	0.2 (5)
C4—C5—C6—C1	−1.0 (5)	C15—C16—C17—O2	−175.3 (3)
C7—N1—C6—C5	−7.6 (5)	C15—C16—C17—C8	2.0 (6)

### Hydrogen-bond geometry (Å, º)

**Table:**

*D*—H···*A*	*D*—H	H···*A*	*D*···*A*	*D*—H···*A*
O3—H3*O*···O1^i^	0.84 (1)	1.80 (1)	2.633 (4)	174 (4)
C2—H2···O2^ii^	0.95	2.50	3.363 (4)	151
C16—H16···Cl3^iii^	0.95	2.74	3.542 (4)	143
C18—H18*A*···Cl2^i^	0.98	2.77	3.707 (4)	161

Symmetry codes: (i) *x*+1/2, −*y*+1/2, −*z*+2; (ii) *x*−1/2, −*y*+1/2, −*z*+2; (iii) *x*, *y*−1, *z*.
